# Neurodevelopmental outcomes in very low birthweight infants with retinopathy of prematurity in a nationwide cohort study

**DOI:** 10.1038/s41598-022-09053-8

**Published:** 2022-03-23

**Authors:** Ja-Hye Ahn, Kyeong Mi Lee, Mi Jung Kim, Hyun-Kyung Park, Yu Jeong Kim, Seong Joon Ahn, Hyun Ju Lee

**Affiliations:** 1 Division of Neonatology and Developmental Medicine, Hanyang University Hospital, Seoul, Republic of Korea; 2 Department of Pediatrics, Hanyang University College of Medicine, 222 Wangsipli-ro, Seongdong-gu, Seoul, 04763 Republic of Korea; 3 Department of Rehabilitation Medicine, Hanyang University College of Medicine, Seoul, Republic of Korea; 4 Department of Ophthalmology, Hanyang University Hospital, Hanyang University College of Medicine, 222 Wangsipli-ro, Seongdong-gu, Seoul, 04763 Republic of Korea

**Keywords:** Eye diseases, Paediatric research

## Abstract

In a nationwide prospective cohort of Korean infants with very low birthweights (VLBW, birth weight < 1500 g) from 70 neonatal intensive care units of the Korean Neonatal Network, we investigated neurodevelopmental outcomes in preterm infants with retinopathy of prematurity (ROP) from 2132 infants with VLBW who had undergone developmental assessments at 18–24 months of corrected age. Motor, cognitive, or language delay was determined using developmental scores that were less than 1 standard deviation from the average. Comparative analyses and multivariate regression analyses were performed to validate the association between ROP or its treatment and developmental delay. Motor (52.8% vs. 36.3%), cognitive (46.8% vs. 31.6%), and language delays (42.5% vs. 28.4%) were noted more frequently in infants with ROP than in those without ROP; this was statistically significant (all P < 0.001). Multivariate analyses showed that motor and cognitive delays were significantly associated with ROP. There were no remarkable differences between the neurodevelopmental outcomes and the treatment modalities (laser photocoagulation, anti-vascular endothelial growth factor injection, or both) for ROP, and both stratification and multivariate regression analyses confirmed no significant association between anti-vascular endothelial growth factor therapy and neurodevelopmental delay. As ROP is significantly associated with poor neurodevelopmental outcomes independent of extreme prematurity, neurodevelopmental functions should be given attention in infants with ROP.

## Introduction

Retinopathy of prematurity (ROP) is caused by vasoproliferation mediated by vascular endothelial growth factor (VEGF), a well-known angiogenic factor^1,2^. This pathological process may lead to structural complications, such as retinal detachment and folding and result in poor visual outcomes despite appropriate treatment. Despite advances in neonatal intensive care and screening/management guidelines, ROP remains one of the leading causes of childhood blindness globally^1,2^.

In addition to the poor visual outcomes, a few reports from retrospective case series showed unfavorable neurodevelopmental outcomes in infants with ROP, particularly in those who were unsuccessfully treated^3–5^. However, other reports showed conflicting results; they found no significant differences in neurodevelopmental functions between those with and without ROP^6,7^. The relationship between neurodevelopmental outcomes and ROP remains unclear, given the small sample size used in previous studies and inconsistent results among the studies; however, this information should be drawn from a larger number of infants to understand developmental disabilities in infants with ROP.

The Korean Neonatal Network (KNN), a national registry of infants with very low birth weight (VLBW, birth weight < 1500 g), was established in 2013 by 70 participating hospitals with governmental support from the Korea Centers for Disease Control and Prevention. Using multicenter clinical data, including ROP details and systemic conditions, prenatal/perinatal factors, maternal factors, systemic or ROP management, and neurodevelopmental assessments, this study aimed to evaluate the neurodevelopmental outcomes in infants with VLBW, with and without ROP. We explored the relationship between ROP and neurodevelopmental outcomes, as well as ROP treatment and outcomes, in a large cohort of Korean VLBW infants.

## Methods

### Study population

The KNN is a nationwide network of infants with VLBW admitted to NICUs of up to 70 participating facilities throughout South Korea. The KNN includes all infants with VLBW, regardless of their gestational age (GA), who were hospitalized or transferred to the neonatal intensive care unit at the participating hospitals within 28 days of birth. All data were prospectively collected according to the guidelines and regulations of the KNN. We reviewed the detailed clinical information of the infants enrolled between January 2013 and December 2017 from the 70 participating NICUs of the KNN registry. Informed consent was obtained from the parents and/or legal guardians of all study participants at enrollment. The Institutional Review Board of Hanyang University Hospital approved our study protocol (IRB no. 2021–02-011).

**Figure 1:**
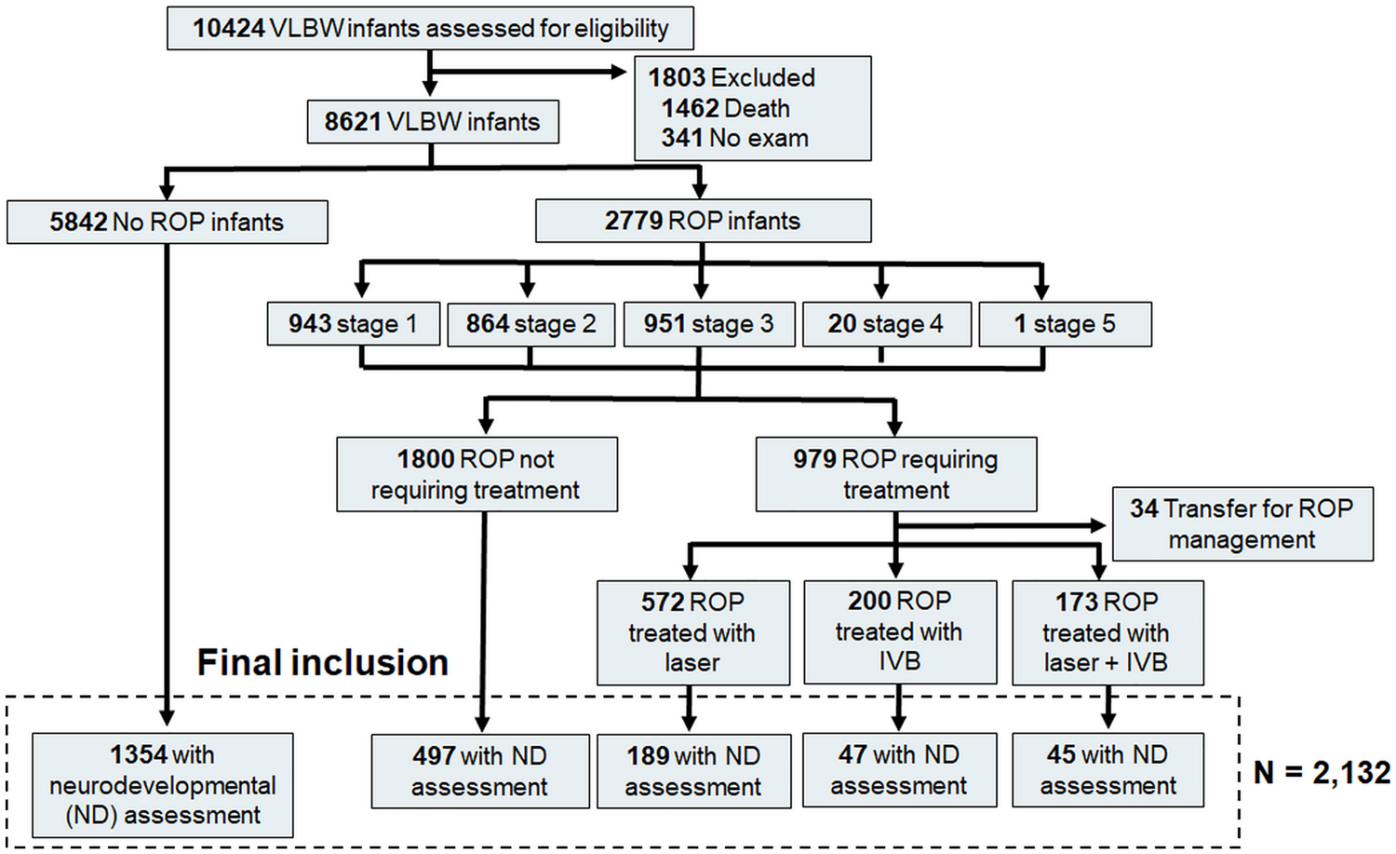
Flowchart illustrating the inclusion and exclusion criteria of the present study and the number of infants meeting the criteria. *VLBW* Very Low birthweight; *ROP* retinopathy of prematurity.

Among the 10,424 infants in the registry, 1803 (17.3%) were excluded because they had not undergone ophthalmic examination (1462 died before the ophthalmic examination). Among the 8621 infants with VLBW, those with neurodevelopmental outcomes evaluated by well-established assessments were included in our analyses. Figure 1 presents a flow diagram of the inclusion and exclusion of our study cohort.

Data on maternal characteristics, neonatal characteristics, and ROP details were collected from the registry. Patients with comorbidities, including necrotizing enterocolitis (NEC, staged according to modified Bell’s staging criteria) and bronchopulmonary dysplasia (BPD, severity determined by the physiologic definition)^8,9^, were also used for our analyses.

The ophthalmic screening for ROP was followed by an established guideline, which recommended the screening of ROP for infants with bodyweights less than 1500 g or GA less than 30 weeks^10^. All the included infants with VLBW underwent binocular indirect ophthalmoscopy by experienced ophthalmologists. The ROP stages were classified from 1 to 5 according to the International Classification of Retinopathy of Prematurity^11–14^. The maximal stage of ROP was recorded in each infant. More detailed information on ROP, including treatment details, was also retrieved from the database. The treatments for ROP included surgery (laser photocoagulation or vitrectomy) and intravitreal injection of anti-vascular endothelial growth factor (anti-VEGF; bevacizumab) agent performed by well-trained ophthalmologists in the participating centers.

We divided the infants into groups based on the presence of ROP (without ROP and with ROP) and treatment (treated and untreated ROP). The treated infants were further separated according to treatment modalities, including laser photocoagulation alone, anti-VEGF therapy alone, and both.

### Neurodevelopmental assessment and patient classification

We analyzed 2132 of 8621 (24.7%) infants with VLBW who had received developmental assessment at 20 months of corrected age on average. Neurodevelopmental outcomes in infants were assessed by the second or third edition of the Bayley Scale of Infant and Toddler Development (BSID) or the Korean Developmental Screening Test (K-DST). The second edition of BSID (BSID-II) consisted of the mental development index and the psychomotor development index, whereas the cognitive, language, and motor scores were obtained using the third edition of the BSID (BSID-III) scale. The raw scores of the BSID scales were converted to standardized scores, with a mean of 100 and a standard deviation of 15. (BSID scores of 100 ± 15 represent mean ± 1 standard deviation [SD]).

The Korean Developmental Screening Test for Infants and Children (K-DST) scores for four domains (gross motor, fine motor, cognition, and language), which had been assessed in several KNN centers where BSID could not be performed, were also retrieved and used for our analyses.

Based on how the neurodevelopmental scores deviated from the average, we determined the presence of neurodevelopmental delay as follows: motor delay as a psychomotor developmental index (PDI) score of < 85 on BSID-II, a motor score of < 85 on BSID-III, or gross or fine motor score of < -1 SD from the average on the K-DST scale; cognitive delay as a mental developmental index (MDI) score of < 85 on BSID-II, a cognitive score of < 85 on BSID-III, or a cognitive score of < -1 SD from the average on the K-DST scale; language delay as a language score of < 85 on BSID-III or < -1 SD from the average on the K-DST scale. As other parameters of neurodevelopmental impairments, data on cerebral palsy and visual impairment were also retrieved. Cerebral palsy was diagnosed using a structured neurological examination, and visual impairment was defined as unilateral or bilateral blindness based on examination by an ophthalmologist. However, the Bayley-III scale may significantly underestimate neurodevelopmental outcomes in those with visual impairment^15^; thus, we excluded the outcomes obtained by BSID-III in those with the impairment (n = 3).

### Data analysis

We compared the maternal and neonatal clinical characteristics and comorbidities of prematurity between infants with and without ROP, as well as among the treatment groups. We also compared the neurodevelopmental outcomes between groups separated based on the presence of ROP and the treatment modalities, including laser photocoagulation, anti-VEGF therapy, and both laser and anti-VEGF therapy. The chi-squared test (categorical variables), Student’s t-test (continuous variables), and analysis of variance (ANOVA; continuous variable) were used to compare the groups. Multivariate regression was used to analyze the associations between the neurodevelopmental outcomes and ROP, as well as those between the outcomes and treatment modalities, by adjusting for other confounding variables, including GA, sex, BPD, intraventricular hemorrhage (IVH), 5-min Apgar score, mechanical ventilation, and supplementary oxygen use. Statistical analyses were performed using SPSS software (version 26.0; SPSS, Chicago, Illinois, USA). P < 0.05 was considered statistically significant.

## Results

### Demographic and clinical characteristics

Of the 8621 infants who underwent ophthalmic examination, 2779 (32.2%) with VLBW were diagnosed with ROP. The infants with ROP consisted of 943 (33.9% of ROP infants), 864 (31.1%), 951 (34.2%), 20 (0.7%), and 1 (0.04%) patients with maximal stages 1, 2, 3, 4, and 5, respectively. Approximately one-third of infants with ROP (979, 35.2%) received ROP treatment, including laser photocoagulation alone (572, 58.4%), anti-VEGF therapy alone (200, 20.4%), or both laser photocoagulation and anti-VEGF therapy (173, 17.7%).

The clinical characteristics of VLBW infants with and without ROP are presented in Table 1. The mean ranges of gestational age were 29.8 ± 2.3 in infants without ROP and 26.7 ± 2.2 weeks in those with ROP; they were statistically significant (*P* < 0.001). The birth weight was 1209.0 ± 209.7 g in the infants without ROP and 934.2 ± 249.6 g in those with ROP (*P* < 0.001). Several maternal characteristics, including pregnancy-induced hypertension (PIH); chorioamnionitis; and neonatal comorbidities, such as respiratory distress syndrome, bronchopulmonary dysplasia, sepsis, necrotizing enterocolitis, and intraventricular hemorrhage, were significantly different between infants with and without ROP. Among the infants with neurodevelopmental assessments, there were also significant differences in the neonatal characteristics and comorbidities between those with and without ROP (*P* < 0.01).

**Table 1:** Demographic characteristics of premature infants with and without retinopathy of prematurity.

Characteristics	Total			Neurodevelopmentally assessed		
	Without ROP (n = 5842)	With ROP (n = 2779)	*P*-value	Without ROP (n = 1354)	With ROP (n = 778)	*P-*value
**Maternal characteristics**						
Maternal PIH	1338/5842 (22.9%)	373/2779 (13.4%)	< 0.001	291/1354 (21.5%)	100/778 (12.9%)	< 0.001
Maternal steroid use	4617/5758 (80.2%)	2266/2723 (83.2%)	0.001	1084/1342 (80.8%)	637/764 (83.4%)	0.137
Chorioamnionitis	1475/4901 (30.1%)	1009/2323 (43.4%)	< 0.001	390/1197 (32.6%)	301/693 (43.4%)	< 0.001
Maternal PROM	1874/5798 (32.3%)	1126/2757 (40.8%)	< 0.001	466/1349 (34.5%)	317/771 (41.1%)	0.003
Maternal GDM	539/5842 (9.2%)	190/2779 (6.8%)	< 0.001	129/1354 (9.5%)	42/778 (5.4%)	0.001
Low maternal education level†	87/4579 (1.9%)	31/2128 (1.5%)	0.199	9/1135 (0.8%)	7/677 (1.0%)	0.596
**Neonatal characteristics and comorbidities**						
Male sex	2942/5842 (50.4%)	1389/2779 (50.0%)	0.743	681/1354 (50.3%)	388/778 (49.9%)	0.851
Gestational age, mean (SD), weeks	29.8 (2.3)	26.7 (2.2)	< 0.001	29.3 (2.3)	26.3 (2.1)	0.007
Birth weight, mean (SD), g	1209.0 (209.7)	934.2 (249.6)	< 0.001	1170.7 (221.9)	890.7 (243.5)	0.043
5-min Apgar score, mean (SD)	7.3 (1.6)	6.4 (1.8)	< 0.001	7.1 (1.5)	6.2 (1.8)	< 0.001
Sepsis	845/5840 (14.5%)	873/2779 (31.4%)	< 0.001	187/1354 (13.8%)	236/778 (30.3%)	< 0.001
NEC stage ≥ 2	175/5839 (3.0%)	256/2779 (9.2%)	< 0.001	36/1353 (2.7%)	76/778 (9.8%)	< 0.001
RDS	4062/5842 (69.5%)	2596/2779 (93.4%)	< 0.001	971/1354 (71.7%)	740/778 (95.1%)	< 0.001
BPD severity ≥ moderate	1120/5786 (19.4%)	1449/2772 (52.3%)	< 0.001	291/1346 (21.6%)	408/777 (52.5%)	< 0.001
Postnatal steroids for BPD	743/5842 (12.7%)	1339/2779 (48.2%)	< 0.001	182/1354 (13.4%)	412/778 (53.0%)	< 0.001
Supplementary oxygen use at 36 weeks of corrected age	979/5793 (16.9%)	1226/2772 (44.2%)	< 0.001	259/1349 (19.2%)	325/777 (41.8%)	< 0.001
Mechanical ventilation at 36 weeks of corrected age	691/5793 (11.9%)	1031/2772 (37.2%)	< 0.001	158/1349 (11.7%)	283/777 (36.4%)	< 0.001
IVH grade ≥ 3	169/5839 (2.9%)	338/2778 (12.2%)	< 0.001	50/1353 (3.7%)	97/778 (12.5%)	< 0.001
PVL	332/5836 (5.7%)	344/2775 (12.4%)	< 0.001	78/1353 (5.8%)	81/777 (10.4%)	< 0.001
PDA	1861/5740 (32.4%)	1682/2691 (62.5%)	< 0.001	492/1343 (36.6%)	514/744 (69.1%)	< 0.001
PDA ligation	346/3918 (8.8%)	582/2200 (26.5%)	< 0.001	84/1343 (6.3%)	168/744 (22.6%)	< 0.001

*ROP* retinopathy of prematurity, *PIH* pregnancy-induced hypertension, *PROM* premature rupture of membranes, *GDM* gestational diabetes mellitus, *RDS* respiratory distress syndrome, *BPD* bronchopulmonary dysplasia, *NEC* necrotizing enterocolitis, *IVH* intraventricular hemorrhage, *PDA* patent ductus arteriosus, *PVL* periventricular leukomalacia.

*For categorical variables, the chi-squared test was used to compare the groups. For continuous variables, unpaired T-test was used to compare the groups.

^†^Low maternal education level indicates less than high school.

The clinical characteristics of the infants with VLBW undergoing laser photocoagulation only, intravitreal anti-VEGF therapy only, and both are shown in Supplementary Table S1. Among the three groups, there were significant differences in gestational age (*P* < 0.001 by ANOVA) and birth weight (*P* = 0.006). There were significant differences in the prevalence of maternal gestational diabetes mellitus (*P* = 0.032), sepsis (*P* = 0.005), necrotizing enterocolitis ≥ stage 2 (*P* = 0.010), intraventricular hemorrhage ≥ grade 3 (*P* < 0.001), and moderate or severe BPD (*P* = 0.030) among the groups.

### Neurodevelopmental outcomes

Neurodevelopmental assessments were performed in 1354 and 778 infants with and without ROP, respectively. As shown in Table 2, motor (52.8% vs. 36.3%), cognitive (46.8% vs. 31.6%), and language delays (42.5% vs. 28.4%) were noted more frequently in infants with ROP than in those without ROP, which was statistically significant (all *P* < 0.001). More specifically, Supplementary Table S2 shows the frequencies of developmental delay in each domain of the K-DST, BSID-II, and BSID-III in infants with and without ROP, showing statistically significant differences in all domains of all scales (all *P* ≤ 0.001). Supplementary Figure S1 shows the linear trends of cognitive, motor, and language delays according to the ROP stage, indicating a severity-dependent association between neurodevelopmental delay and ROP.

**Table 2:** Neurodevelopmental outcomes in infants with and without retinopathy of prematurity (ROP) among those with assessed neurodevelopmental outcomes.

Outcomes	Without ROP No. of infants/no. of assessed	With ROP No. of infants/no. of assessed	*P*-value*
Motor delay^†^	491/1352 (36.3%)	410/777 (52.8%)	< 0.001
Cognitive delay^†^	427/1352 (31.6%)	364/777 (46.8%)	< 0.001
Language delay^†^	329/1160 (28.4%)	269/633 (42.5%)	< 0.001
Cerebral palsy	63/1327 (4.7%)	78/753 (10.4%)	< 0.001
Hemiplegia or severer	28/1327 (2.1%)	34/753 (4.5%)	0.002

*Chi-square test or Fisher’s exact test.

^†^Motor delay is represented by a PDI score of < 85 on BSID-II or a motor score of < 85 on BSID-III or < − 1 SD on K-DST scales. Cognitive delay is represented by an MDI score of < 85 on the BSID-II subsets or a cognitive score of < 85 on the BSID-III or < − 1 SD on the K-DST scales. Language delay is represented by a language score of < 85 on the BSID-III or < − 1 SD on the K-DST scales. As the Bayley-III scale may significantly underestimate neurodevelopmental outcomes in those with visual impairment, we have excluded the outcomes obtained by BSID-III in those with the impairment (n = 3).

The prevalence of cerebral palsy (4.7% and 10.4%) was significantly different between the groups (*P* < 0.001), indicating that these impairments were more frequently noted in VLBW infants with ROP than in those without ROP.

### Association between ROP and neurodevelopmental outcomes

The results of univariate and multivariate logistic regression analyses based on the factors associated with neurodevelopmental outcomes are presented in Table 3. In multivariate analyses using the factors significantly associated with neurodevelopmental delay as covariates, ROP was significantly associated with motor (odds ratio [OR] = 1.37 [95% confidence interval (CI) 1.10–1.72], *P* = 0.005) and cognitive delays (OR = 1.36 [95% CI 1.09–1.71], *P* = 0.008) when adjusted for maternal and perinatal factors, such as maternal PIH, sex, GA, IVH, BPD, 5-min Apgar score, and mechanical ventilation.

**Table 3:** Clinical factors associated with developmental delay in univariate and multivariate regression analyses.

	Motor delay*				Cognitive delay*				Language delay*			
	Univariate		Multivariate		Univariate		Multivariate		Univariate		Multivariate	
	OR (95% CI)	*P*	OR (95% CI)	*P*	OR (95% CI)	*P*	OR (95% CI)	*P*	OR (95% CI)	*P*	OR (95% CI)	*P*
Maternal PIH	0.77 (0.61–0.97)	0.023	0.94 (0.74–1.19)	0.594	0.70 (0.55–0.88)	0.003	0.86 (0.67–1.10)	0.220	0.90 (0.69–1.16)	0.404	N/A	N/A
Sex, male to female	1.35 (1.14–1.61)	0.001	1.31 (1.10–1.57)	0.003	1.72 (1.44–2.06)	< 0.001	1.69 (1.41–2.03)	< 0.001	1.77 (1.45–2.15)	< 0.001	1.76 (1.43–2.16)	< 0.001
Gestational age, mean (SD), weeks^†^	0.89 (0.86–0.92)	< 0.001	0.96 (0.92–1.01)	0.079	0.88 (0.85–0.91)	< 0.001	0.95 (0.91–1.00)	0.037	0.88 (0.85–0.92)	< 0.001	0.95 (0.90–1.00)	0.053
Birth weight, mean (SD), g^†^	1.00 (1.00–1.00)	< 0.001	N/A	N/A	1.00 (1.00–1.00)	< 0.001	N/A	N/A	1.00 (1.00–1.00)	< 0.001	N/A	N/A
5-min Apgar score	0.89 (0.84–0.94)	< 0.001	0.97 (0.92–1.03)	0.293	0.90 (0.85–0.94	< 0.001	0.98 (0.92–1.04)	0.484	0.89 (0.84–0.94)	< 0.001	0.98 (0.92–1.05)	0.607
BPD severity ≥ moderate	1.49 (1.20–1.85)	< 0.01	1.14 (0.87–1.49)	0.335	1.58 (1.27–1.97)	< 0.01	1.16 (0.88–1.52)	0.295	1.80 (1.41–2.29)	< 0.01	1.12 (0.82–1.53)	0.474
Supplementary oxygen use at 36 weeks of corrected age^†^	1.67 (1.38–2.02)	< 0.001	N/A	N/A	1.40 (1.15–1.69)	0.001	N/A	N/A	1.99 (1.61–2.48)	< 0.001	N/A	N/A
Mechanical ventilation at 36 weeks of corrected age^†^	2.18 (1.76–2.70)	< 0.001	1.49 (1.11–2.01)	0.008	1.98 (1.60–2.45)	< 0.001	1.35 (1.00–1.81)	0.050	3.07 (2.42–3.89)	< 0.001	2.25 (1.61–3.15)	< 0.001
IVH grade ≥ 3	3.69 (2.55–5.34)	< 0.001	2.70 (1.83–3.97)	< 0.001	2.60 (1.85–3.66)	< 0.001	1.82 (1.27–2.62)	0.001	2.74 (1.90–3.95)	< 0.001	1.83 (1.24–2.71)	0.002
ROP	1.96 (1.64–2.34)	< 0.001	1.37 (1.10–1.72)	0.005	1.91 (1.59–2.29)	< 0.001	1.36 (1.09–1.71)	0.008	1.87 (1.52–2.29)	< 0.001	1.20 (0.93–1.56)	0.164

*OR* odds ratio, *CI* confidence interval, *ROP* retinopathy of prematurity, *PIH* pregnancy-induced hypertension, *PROM* premature rupture of membranes, *GDM* gestational diabetes mellitus, *RDS* respiratory distress syndrome, *BPD* bronchopulmonary dysplasia, *NEC* necrotizing enterocolitis, *IVH* intraventricular hemorrhage, *PDA* patent ductus arteriosus, *PVL* periventricular leukomalacia, *BSID* Bayley scale of infant and Toddler Development, *K-DST* Korean Developmental Screening Test, *SD* standard deviation, *N/A* not applicable.

*Motor delay is represented by a PDI score of < 85 on BSID-II subsets, a motor score of < 85 on BSID-III subsets, or a motor score of < − 1 SD on K-DST subsets. Cognitive delay is represented by an MDI score of < 85 on BSID II subsets, a cognitive score of < 85 on BSID-III subsets or < − 1 SD on K-DST subsets. Language delay is represented by a language score of < 85 on BSID-III subsets or < − 1 SD on K-DST subsets.

^†^The variables with significant correlation were not analyzed simultaneously to avoid multicollinearity (birth weights and mechanical ventilation at 36 weeks of were not used due to significant correlation with gestational age and supplementary oxygen use at the time).

Table 4 shows the comparison of neurodevelopmental outcomes in the ROP groups separated by treatment modalities. Ninety-six (51.1%), 16 (34.0%), and 30 (66.7%) infants receiving treatment with laser treatment, anti-VEGF therapy, and both laser and anti-VEGF, respectively, had cognitive delays, which was significantly different. (*P* = 0.007) However, the motor or language delays did not significantly differ among the three groups (both *P* > 0.05). Cerebral palsy was not significantly different among the groups, whereas visual impairment was more frequently noted in infants treated with both laser and anti-VEGF treatment. (*P* < 0.001).

**Table 4:** Neurodevelopmental outcomes in infants treated for retinopathy of prematurity (ROP).

Outcomes	Laser only, No./no. of assessed	Anti-VEGF only, No./no. of assessed	Both, no./no. of assessed	*P*-value*
Motor delay^†^	106/188 (56.4%)	30/47 (63.8%)	32/45 (71.1%)	0.163
Cognitive delay^†^	96/188 (51.1%)	16/47 (34.0%)	30/45 (66.7%)	0.007
Language delay^†^	80/168 (47.6%)	11/26 (42.3%)	19/38 (50.0%)	0.828
Cerebral palsy	24/185 (13.0%)	11/46 (23.9%)	7/44 (15.9%)	0.181
Hemiplegia or severer	10/185 (5.4%)	4/46 (8.7%)	5/44 (11.4%)	0.241

*Chi-square test or Fisher’s exact test.

^†^Motor delay is represented by a PDI score of < 85 on BSID-II or a motor score of < 85 on BSID-III or < − 1 SD on K-DST scales. Cognitive delay is represented by an MDI score of < 85 on the BSID-II subsets or a cognitive score of < 85 on the BSID-III or < − 1 SD on the K-DST scales. Language delay is defined as a language score of < 85 on the BSID-III or < − 1 SD on the K-DST scales.

Supplementary Table S3 presents the frequencies of motor, cognitive, and language delay in subgroups separated based on the zone of ROP at the treatment, zone 1 and other ROP. In each stratum of zone 1 and zone 2 or 3 ROP, the prevalence of each neurodevelopmental delay was not significantly different among the treatment groups. However, cerebral palsy was significantly different among the treatment groups in infants with zone 2 or 3 ROP. The multivariate logistic analyses among the infants with ROP, presented in Supplementary Table S4, indicated that anti-VEGF therapy was not significantly associated with any neurodevelopmental delay (all *P* > 0.05).

## Discussion

From the data obtained from a prospective registry of Korean infants with VLBW, the present study showed that ROP was associated with poor neurodevelopmental outcomes in infants with VLBW. Specifically, those with ROP showed more frequent cognitive, motor, and language delays than those without ROP.

As ROP is usually associated with comorbidities, including extreme prematurity and IVH, the confounding effects of these comorbidities should be carefully adjusted to evaluate an independent association between ROP and neurodevelopmental outcomes. In the present study, several findings support the association between ROP and poor neurodevelopmental outcomes. First, the significant difference in neurodevelopmental outcomes between those with and without ROP and linear relationship between ROP stages and neurodevelopmental outcomes (Supplementary Fig. S1) suggests an association between ROP and neurodevelopmental delay. In addition, multivariate analyses involving GA and other factors potentially affecting brain development as confounders showed that ROP was significantly associated with cognitive and motor delays in infants with ROP, which was independent of prematurity-associated factors.

In previous studies, neurodevelopmental delay was associated with the degree of prematurity^16^. The delay may also result from ROP, particularly zone I ROP^16^, although there have been conflicting reports on the association between ROP and neurodevelopmental outcomes between studies^6,9,17–20^. For example, a few prospective case–control study showed that there were no significant differences in the neurodevelopmental outcomes between infants with and without ROP^6,7^. However, several authors have suggested that ROP or its severe type is associated with a poor prognosis of child neurodevelopment^5,16^.

In addition, there have been conflicting results on the association between neurodevelopmental functions and ROP treatment modalities. The Bevacizumab Eliminates the Angiogenic Threat of ROP (BEAT-ROP) trial demonstrated favorable outcomes of intravitreal anti-vascular endothelial growth factor (VEGF) therapy for type 1 ROP^21^. Subsequently, anti-VEGF therapy, with or without conventional laser photocoagulation, has been used as an alternative treatment for treatment-requiring ROP. Despite the better outcomes of refractive errors or visual field defects and favorable foveal development in infants treated with anti-VEGF therapy, some concerns regarding systemic safety for the use of therapy in preterm infants exist, as VEGF is also a key molecule for CNS development; thus, anti-VEGF therapy may affect neurodevelopment. Some studies have shown that there are no significant differences in severe neurodevelopmental impairment in bevacizumab-treated infants compared with laser-treated infants^9,19^ or untreated infants^6,18^. In-depth analyses of cognitive, language, and motor functions showed no significant differences between ROP infants treated with bevacizumab and laser therapy or untreated controls^6,9,17–19^. However, Morin et al. reported a conflicting result; they observed a lower motor score and a higher rate of severe neurodevelopmental impairment in infants with anti-VEGF-treated ROP than in those with laser-treated ROP^20^. A recent meta-analysis based on current evidence showed that the risk of severe neurodevelopmental impairment did not increase, but there was a minor difference in motor performance on the Bayley III scales in the infants treated with bevacizumab therapy^22^. The results obtained from our large number of infants were consistent with the aforementioned results, as infants with ROP had poor neurodevelopmental outcomes, but most of the outcomes were comparable among the treatment groups. Furthermore, both stratification and multivariate analyses for controlling potential confounders in our study revealed no significant association between anti-VEGF therapy and neurodevelopmental delay in motor, cognitive, and language domains.

Several nationwide neonatal research networks have been introduced, including the National Institute of Child Health and Human Development Neonatal Research Network (NICHD NRN) in the United States, the Canadian Neonatal Network (CNN), and the Neonatal Research Network of Japan (NRNJ)^23^. Similar to the above networks, the KNN is established to facilitate neonatal research and improvement in the quality of neonatal care, which would lead to improved developmental prognoses of the infants with VLBW. Because ROP is caused by abnormal development of retinal blood vessels and the retina is part of the CNS, neurodevelopmental outcomes may have to be carefully evaluated for developmental and functional prognoses in infants with VLBW with ROP. In light of this, our results suggest that attention should be paid to neurodevelopmental function in infants with VLBW and ROP, based on the findings of poor neurodevelopmental outcomes obtained from a large number of infants.

The strengths of this study include the large database incorporating comprehensive clinical and neurodevelopmental data from multiple sites across the country. However, this study has some limitations that require careful interpretation of our results. First, the relatively short-term (20 months) results for neurodevelopmental outcomes cannot be extrapolated to long-term results, as CNS development and neurodevelopmental functions in children are changing constantly, and our findings can be very dynamic. Accordingly, these results should be interpreted with great care for neurodevelopment in children with ROP. Second, among the 8621 infants who underwent ophthalmic examinations, only 2132 (24.7%) with available neurodevelopmental assessments were analyzed. This was mainly due to a follow-up loss of approximately 30% at ages of 18–24 months^24^. The unavailability of neurodevelopmental data may have also resulted from the absence of specialized child psychologists in some participating centers. Furthermore, our data on the frequencies of neurodevelopmental and visual outcomes in the severity subgroups, particularly those for zone 1 ROP, are limited by the small number of infants in each group. Third, the heterogeneity of neurodevelopmental assessments, including BSID-II/III and K-DST, may have introduced bias in our analyses. For example, language delay could not be determined in the children assessed by BSID-II, as Bayley II reports mental and psychomotor developmental indices only. Additionally, several studies have shown that the BSID-III scores can over- or underestimate infant performance compared to the BSID-II scores^25,26^. Therefore, we did not use individual neurodevelopmental scores but used the presence or absence of neurodevelopmental delay determined by standardized scores for each infant, which might reduce bias occurring when combining the data obtained by different assessments. Fourth, we could not control the diagnosis and treatment of ROP of the individual participating centers, which may be diverse and potentially lead to bias. In addition, despite our efforts to minimize the potential confounding effects of several clinical factors and ROP details on neurodevelopmental outcomes, we could not completely exclude the confounding effects of various factors. Therefore, systemic conditions and other perinatal factors should be carefully adjusted to draw conclusions on the neurodevelopmental outcomes in infants born with VLBW. Additionally, a previous study showed that the accommodations of BSID-III for visually deprived infants improved their cognition scale test scores^15^. Although our study was designed to apply the most established and widely used assessment to minimize inter-center variability, such adaptations may better reflect the neurodevelopmental outcomes of visually deprived infants. Finally, although this study included most Korean infants with VLBW, up to 30% were not represented in this study, as the KNN covers 70–80% of Korean infants with VLBW born each year in Korea^24^.

In conclusion, our findings suggest that ROP may be associated with poor neurodevelopmental outcomes in infants born prematurely. Specifically, ROP was significantly associated with motor and cognitive delays. These findings highlight the importance of neurodevelopmental assessment and monitoring in conjunction with ophthalmic monitoring in infants with ROP.

## Supplementary Information

Supplementary Information.
