# Supplementary material for: Neurodevelopmental outcomes in very low birthweight infants with retinopathy of prematurity in a nationwide cohort study
Source: Sci Rep. 2022 Mar 23;12:5053. doi: 10.1038/s41598-022-09053-8 (PMC8943194; doi:10.1038/s41598-022-09053-8)
Supplement: Supplementary file 1 — Supplementary Information. [file 41598_2022_9053_MOESM1_ESM.docx]

**Neurodevelopmental Outcomes in Very Low Birthweight Infants with Retinopathy of Prematurity in a Nationwide Cohort Study**

Ja Hye Ahn^1,2*^, Kyeong Mi Lee^1,2^*, Mi Jung Kim^3^, Hyun-Kyung Park^1,2^, Yu Jeong Kim^4^, Seong Joon Ahn^4^, Hyun Ju Lee^1,2^, on behalf of the Korean Neonatal Network

^1^Division of Neonatology and Developmental Medicine, Hanyang University Hospital, Seoul, Republic of Korea

^2^Department of Pediatrics, Hanyang University Hospital, Hanyang University College of Medicine, Seoul, Republic of Korea

^3^Department of Rehabilitation Medicine, Hanyang University College of Medicine, Seoul, Republic of Korea

^4^Department of Ophthalmology, Hanyang University Hospital, Hanyang University College of Medicine, Seoul, Republic of Korea

*Ja Hye Ahn and Kyeong Mi Lee contributed equally to this work.

*Corresponding Authors: Seong Joon Ahn, MD, PhD, [ahnsj81@gmail.com](mailto:ahnsj81@gmail.com), Hyun Ju Lee, MD, PhD, [blesslee77@hanmail.net](mailto:blesslee77@hanmail.net)

**Supplementary Table S1.** Demographic characteristics of the infants treated for retinopathy of prematurity

| **Characteristics** | **Laser alone (n=572)** | **Anti-VEGF alone (n=200)** | **Both (n=173)** | ***P*-value*** |
| --- | --- | --- | --- | --- |
| ***Maternal characteristics*** |  | | | |
| Maternal PIH | 62/572 (10.8%) | 23/200 (11.5%) | 22/173 (12.7%) | 0.789 |
| Maternal steroid use | 464/558 (83.2%) | 160/195 (82.1%) | 145/170 (85.3%) | 0.700 |
| Chorioamnionitis | 251/487 (51.5%) | 80/177 (45.2%) | 80/155 (51.6%) | 0.325 |
| Maternal PROM | 229/565 (40.5%) | 91/198 (46.0%) | 64/170 (37.6%) | 0.241 |
| Maternal GDM | 33/572 (5.8%) | 10/200 (5.0%) | 19/173 (11.0%) | **0.032** |
| Low maternal education level^†^ | 4/454 (0.9%) | 0/165 (0.0%) | 2/137 (1.5%) | 0.358 |
| ***Neonatal characteristics*** |  | | | |
| Male sex | 296/572 (51.7%) | 97/200 (48.5%) | 93/173 (53.8%) | 0.581 |
| Gestational age, mean (SD), weeks | 25.6 (1.8) | 25.8 (2.2) | 25.0 (1.8) | **<0.001** |
| Birth weight, mean (SD), g | 826.7 (216.0) | 831.7 (230.2) | 768.6 (219.0) | **0.006** |
| 5-min Apgar score, mean (SD) | 5.9 (1.8) | 5.9 (1.8) | 5.8 (1.9) | 0.870 |
| ***Neonatal comorbidity*** |  | | | |
| Sepsis | 238/572 (41.6%) | 65/200 (32.5%) | 85/173 (49.1%) | **0.005** |
| NEC stage ≥ 2 | 72/572 (12.6%) | 22/200 (11.0%) | 36/173 (20.8%) | **0.010** |
| RDS | 556/572 (97.2%) | 191/200 (95.5%) | 167/173 (96.5%) | 0.502 |
| BPD severity ≥ moderate | 381/572 (66.6%) | 138/200 (69.0%) | 133/172 (77.3%) | **0.029** |
| Postnatal steroids for BPD | 343/572 (60.0%) | 118/200 (59.0%) | 115/173 (66.5%) | 0.250 |
| Supplementary oxygen use at 36 weeks^‡^ | 323/571 (56.6%) | 128/200 (64.0%) | 100/172 (58.1%) | 0.185 |
| Mechanical ventilation at 36 weeks^‡^ | 295/571 (51.7%) | 95/200 (47.5%) | 119/172 (69.2%) | **<0.001** |
| IVH grade ≥ 3 | 86/572 (15.0%) | 51/200 (25.5%) | 44/173 (25.4%) | **<0.001** |
| PVL | 74/569 (13.0%) | 37/200 (18.5%) | 31/173 (17.9%) | 0.089 |
| PDA | 426/559 (76.2%) | 132/188 (70.2%) | 131/170 (77.1%) | 0.210 |
| PDA ligation | 185/487 (38.0%) | 60/148 (40.5%) | 67/144 (46.5%) | 0.183 |

Abbreviations : VEGF, vascular endothelial growth factor; PIH, pregnancy-induced hypertension; PROM, premature rupture of membranes; GDM, gestational diabetes mellitus; RDS, respiratory distress syndrome; BPD, bronchopulmonary dysplasia; NEC, necrotizing enterocolitis; IVH, intraventricular hemorrhage; PDA, patent ductus arteriosus; PVL, periventricular leukomalacia.

^*^For categorical variables, the chi-squared test was used for comparison between groups. For continuous variables, analysis of variance was used to compare the groups.

^†^Low maternal education level is less than high school.

^‡^Corrected age

**Supplementary Table S2**. Outcomes of subsets of evaluated neurodevelopmental assessments in infants with and without retinopathy of prematurity

| **Developmental assessments** | **Without ROP**  **No. of infants / No. of assessed** | | **With ROP**  **No. of infants / No. of assessed** | ***P*-value*** |
| --- | --- | --- | --- | --- |
| **K-DST** |  |  | |  |
| Gross motor (<-1SD) | 275 / 917 (30.0%) | 228 / 511 (44.6%) | | **<0.001** |
| Fine motor (<-1SD) | 225 / 917 (24.5%) | 184 / 511 (36.0%) | | **<0.001** |
| Cognition (<-1SD) | 283 / 917 (30.9%) | 201 / 511 (39.3%) | | **0.001** |
| Language (<-1SD) | 263 / 917 (28.7%) | 221 / 511 (43.2%) | | **<0.001** |
| **BSID-II** |  |  | |  |
| MDI (<-1SD) | 170 / 434(39.2%) | 205 / 378 (54.2%) | | **<0.001** |
| PDI (<-1SD) | 167 / 434(38.5%) | 189 / 378 (50.0%) | | **0.001** |
| **BSID-III** |  |  | |  |
| Motor (<-1SD) | 44 / 337 (13.1%) | 61 / 174 (35.1%) | | **<0.001** |
| Cognitive (<-1SD) | 35 / 337 (10.4%) | 47 / 174 (27.0%) | | **<0.001** |
| Language (<-1SD) | 75 / 337(22.3%) | 70 / 174 (40.2%) | | **<0.001** |

Abbreviations: ROP, retinopathy of prematurity; K-DST, Korean Developmental Screening Test; BSID, Bayley Scales of Infant and Toddler Development

^*^For categorical variables, the chi-squared test was used to compare the groups.

**Supplementary Table S3.** Neurodevelopmental outcomes in subgroups of zone of retinopathy of prematurity (ROP) among infants treated for ROP

| **Outcomes** | **Zone 1 ROP** | | | | **Other ROP** | | | |
| --- | --- | --- | --- | --- | --- | --- | --- | --- |
|  | **Laser only** | **Anti-VEGF only** | **Both** | ***P*-value*** | **Laser only** | **Anti-VEGF only** | **Both** | ***P*-value*** |
| **Motor delay**^†^ | 25.0% | 62.5% | 100% | 0.131 | 87.5% | 75.0% | 66.7% | 0.594 |
| **Cognitive delay**^†^ | 25.0% | 62.5% | 100% | 0.131 | 37.5% | 12.5% | 66.7% | 0.203 |
| **Language delay**^†^ | 75.0% | 50.0% | 100% | 0.269 | 42.9% | 33.3% | 50.0% | 0.928 |
| **Cerebral palsy** | 25.0% | 25.0% | 100% | 0.060 | 0 | 37.5% | 33.3% | **0.032** |
| Hemiplegia or severer | 0 | 12.5% | 33.3% | 0.436 | 0 | 12.5% | 33.3% | 0.104 |
| **Visual impairment** | 0 | 12.5% | 33.3% | 0.436 | 0 | 0 | 0 | N/A |

N/A = not applicable

* Chi-square test or Fisher’s exact test was used for statistical analyses.

**Supplementary Table S4.** Clinical factors associated with developmental delay in univariate and multivariate regression analyses among infants treated for retinopathy of prematurity

|  | **Motor delay*** | | | | **Cognitive delay*** | | | | **Language delay*** | | | |
| --- | --- | --- | --- | --- | --- | --- | --- | --- | --- | --- | --- | --- |
|  | **Univariate** |  | **Multivariate** |  | **Univariate** |  | **Multivariate** |  | **Univariate** |  | **Multivariate** |  |
|  | **OR (95% CI)** | ***P*** | **OR (95% CI)** | ***P*** | **OR (95% CI)** | ***P*** | **OR (95% CI)** | ***P*** | **OR (95% CI)** | ***P*** | **OR (95% CI)** | ***P*** |
| Male sex^†^ | 2.35 (1.44-3.84) | **0.001** | 2.16 (1.29-3.62) | **0.004** | 1.65 (1.03-2.64) | **0.038** | 1.41 (0.86-2.33) | 0.178 | 1.13 (0.67-1.89) | 0.648 | 0.99 (0.57-1.72) | 0.974 |
| Gestational age, mean (SD), weeks^†^ | 0.85 (0.75-0.98) | **0.025** | 0.93 (0.80-1.09) | 0.372 | 0.79 (0.69-0.91) | **0.001** | 0.88 (0.76-1.03) | 0.107 | 0.89 (0.76-1.03) | 0.125 | 1.01 (0..85-1.20) | 0.941 |
| Birth weight, mean (SD), g^†^ | 1.00 (1.00-1.00) | 0.066 | N/A | N/A | 1.00 (1.00-1.00) | **0.001** | N/A | N/A | 1.00 (1.00-1.00) | **0.001** | N/A | N/A |
| 5-min Apgar score | 1.01 (0.89-1.15) | 0.838 | 1.06 (0.92-1.23) | 0.397 | 0.92 (0.81-1.05) | 0.202 | 0.96 (0.83-1.10) | 0.525 | 0.88 (.076-1.01) | 0.060 | 0.87 (0.75-1.02) | 0.083 |
| BPD severity ≥ moderate | 1.72 (1.03-2.86) | **0.038** | 1.30 (0.58-2.93) | 0.527 | 2.06 (1.23-3.45) | **0.006** | 1.00 (0.45-2.23) | 0.992 | 2.16 (1.23-3.80) | **0.007** | 0.75 (0.31-1.85) | 0.533 |
| Supplementary oxygen use at 36 weeks of corrected age^†^ | 1.35 (0.83-21.8) | 0.223 | N/A | N/A | 1.60 (1.00-2.57) | 0.052 | N/A | N/A | 1.93 (1.14-3.25) | **0.014** | N/A | N/A |
| Mechanical ventilation at 36 weeks of corrected age^†^ | 1.65 (1.02-2.67) | **0.041** | 1.09 (0.52-2.30) | 0.818 | 2.27 (1.41-3.67) | **0.001** | 1.79 (0.87-3.66) | 0.113 | 2.75 (1.62-4.68) | **<0.001** | 2.97 (1.32-6.70) | **0.009** |
| IVH grade ≥ 3 | 7.77 (2.98-20.27) | **<0.001** | 6.35 (2.37-17.05) | **<0.001** | 4.33 (2.11-8.87) | **<0.001** | 3.21 (1.52-6.77) | **0.002** | 3.09 (1.49-6.44) | **0.003** | 2.75 (1.26-5.99) | **0.011** |
| Anti-VEGF therapy^†^ | 1.58 (0.94-2.67) | 0.084 | 1.57 (0.89-2.75) | 0.118 | 0.95 (0.58-1.56) | 0.835 | 0.86 (0.50-1.47) | 0.573 | 0.96 (0.54-1.71) | 0.886 | 0.89 (0.48-1.65) | 0.710 |
| Zone 1 ROP^†^ | 0.34 (0.08-1.41) | 0.137 | N/A | N/A | 3.00 (0.81-11.08) | 0.099 | N/A | N/A | 2.75 (0.67-11.24) | 0.159 | N/A | N/A |

Abbreviations: OR, odds ratio; CI, confidence interval; ROP, retinopathy of prematurity; PIH, pregnancy-induced hypertension; PROM, premature rupture of membranes; GDM, gestational diabetes mellitus; RDS, respiratory distress syndrome; BPD, bronchopulmonary dysplasia; NEC, necrotizing enterocolitis; IVH, intraventricular hemorrhage; PDA, patent ductus arteriosus; PVL, periventricular leukomalacia; BSID, Bayley scale of infant and Toddler Development; K-DST, Korean Developmental Screening Test; SD, standard deviation

*Motor delay is represented by a PDI score of < 85 on BSID II subsets or a motor score of < 85 on BSID III subsets or < -1 SD on K-DST subsets. Cognitive delay is represented by an MDI score of < 85 on the BSID II subsets or a cognitive score of < 85 on the BSID III subsets or < -1 SD on the K-DST subsets. Language delay is represented by a language score of < 85 on the BSID III subsets or < -1 SD on the K-DST subsets.

^†^The variables with significant correlation were not analyzed simultaneously to avoid multicollinearity (birth weight, mechanical ventilation at 36 weeks of corrected age, and zone 1 ROP were not used due to significant correlation with gestational age, supplementary oxygen use, and anti-VEGF therapy, respectively).

**Supplementary Figure S1.** Neurodevelopmental outcomes stratified by the stages of retinopathy of prematurity
